# Supplementary material for: All-in-one porous membrane enables full protection in guided bone regeneration
Source: Nat Commun. 2024 Jan 2;15:119. doi: 10.1038/s41467-023-43476-9 (PMC10762214; doi:10.1038/s41467-023-43476-9)
Supplement: Supplementary file 1 — Supplementary information [file 41467_2023_43476_MOESM1_ESM.pdf]

Supplementary Information

All-in-one asymmetric porous membrane enables full protection in guided bone regeneration

Shuyi Wu[1, #], Shulu Luo [1, #], Zongheng Cen[2, #], Qianqian Li[1], Luwei Li[1], Weiran Li[1], Zhike Huang[3], Wenyi He[2], Guobin Liang[1], Dingcai Wu[2, *], Minghong Zhou[3, *] & Yan Li[1, *]

[1] Hospital of Stomatology, Guanghua School of Stomatology, Guangdong Provincial Key Laboratory of Stomatology, Sun Yat-sen University, Guangzhou 510055, P. R. China.

[2] Key Laboratory for Polymeric Composite and Functional Materials of Ministry of Education, School of Chemistry, Sun Yat-sen University, Guangzhou 510006, P.R. China.

[3] Medical Research Institute, Guangdong Provincial People's Hospital (Guangdong Academy of Medical Sciences), Southern Medical University, Guangzhou 510080, P. R. China.

[#] These authors contributed equally: Shuyi Wu, Shulu Luo, Zongheng Cen.

* Corresponding author’s email: liy8@mail.sysu.edu.cn; zhouminghong@gdph.org.cn; wudc@mail.sysu.edu.cn

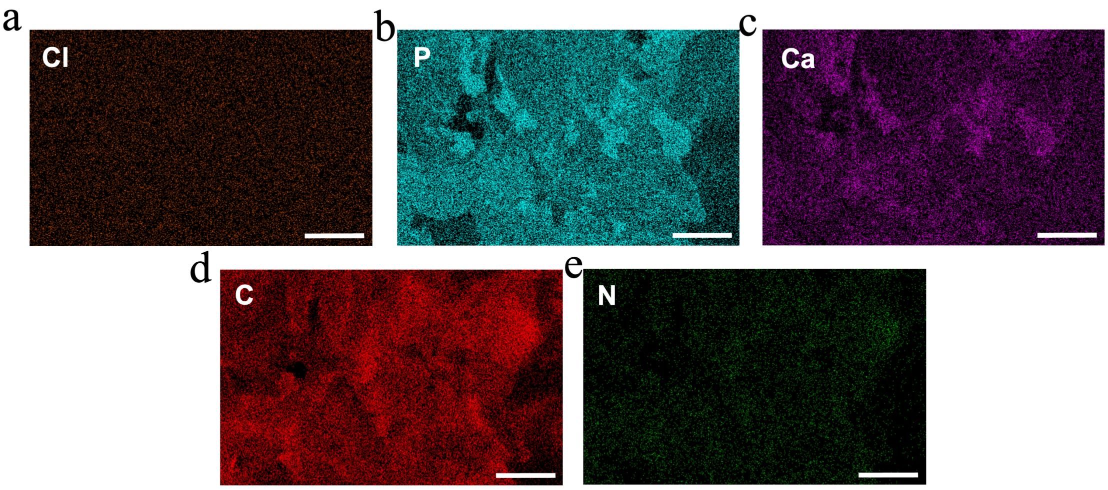

Supplementary Fig. 1 Elemental distributions of BC-*g*-NCl/CS-HAP membrane. **a** Elemental mapping image of Cl **(a)** in the BC-*g*-NCl layer (scale bar = 2 μm), showing Cl element was evenly introduced in BC-*g*-NCl layer. **b-e** Elemental mapping images of P **(b)**, Ca **(c),** C **(d)** and N **(e)** in the CS-HAP layer (scale bars = 10 μm), showing HAP particles and CS were well composited in CS-HAP layer. Observation was repeated three times independently, yielding similar results.

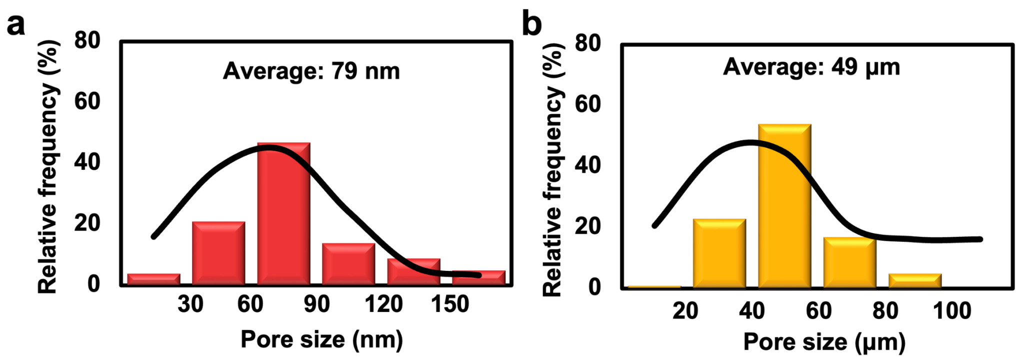

Supplementary Fig. 2 Pore size distributions of the dense and loose layers. a, b Pore size distributions of BC-*g*-PNCl dense layer **(a)** and CS-HAP loose layer **(b)** of BC-*g*-PNCl/CS-HAP according to the analyses of SEM images in Fig. 2e, f.

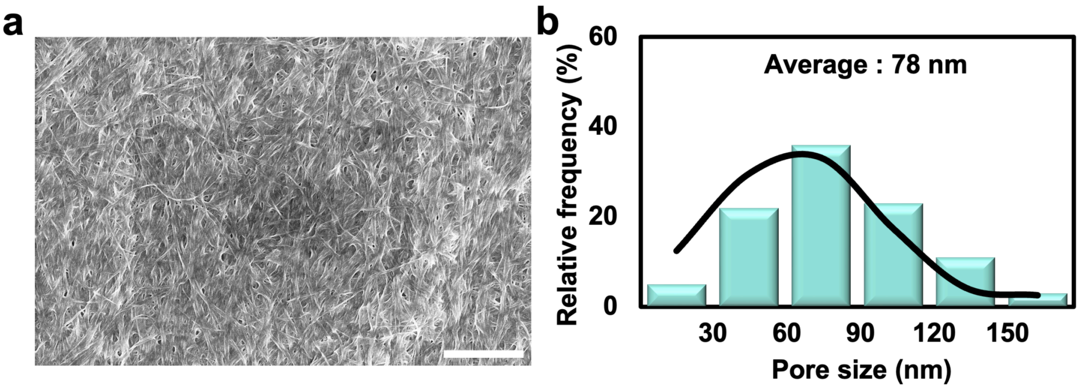

Supplementary Fig. 3 Surface morphology and pore size distribution of BC. a, b SEM image **(a)** (scale bar = 2 μm) and corresponding pore size distribution **(b)** of BC. SEM observation was repeated three times independently, yielding similar results.

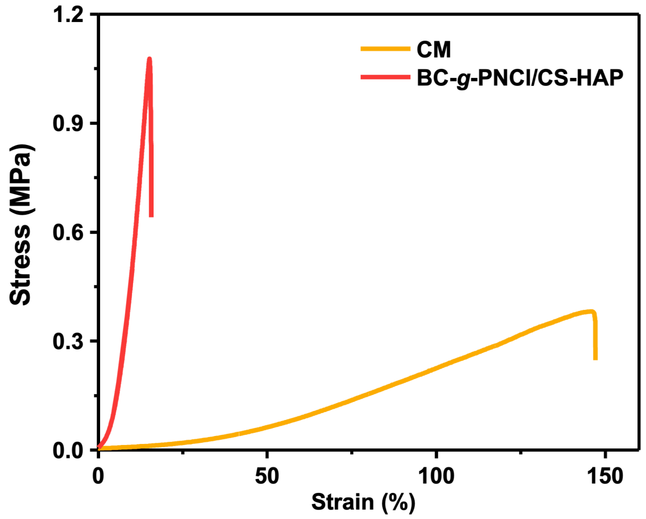

Supplementary Fig. 4 Tensile stress-strain curves of CM and BC-*g*-PNCl/CS-HAP in the wet state.

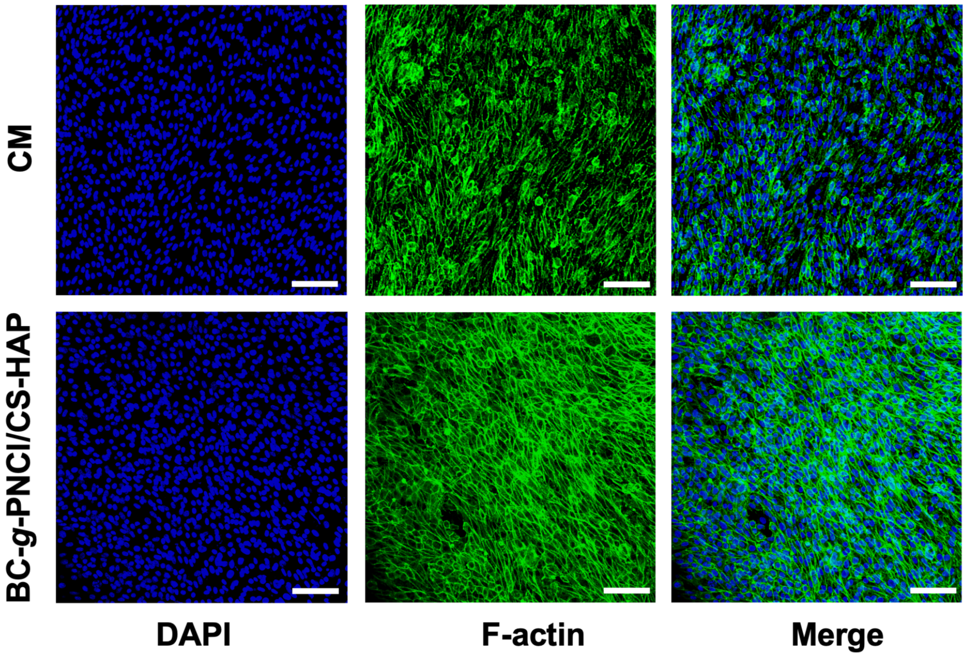

Supplementary Fig. 5 Morphology, extension, and viability of L929s. Morphology of L929s cultured on dense layer of CM and BC-*g*-PNCl layer of BC-*g*-PNCl/CS-HAP at the 7th day (green for F-actin, blue for cell nucleus, scale bars = 100 μm). Fluorescent observation was repeated three times independently, yielding similar results.

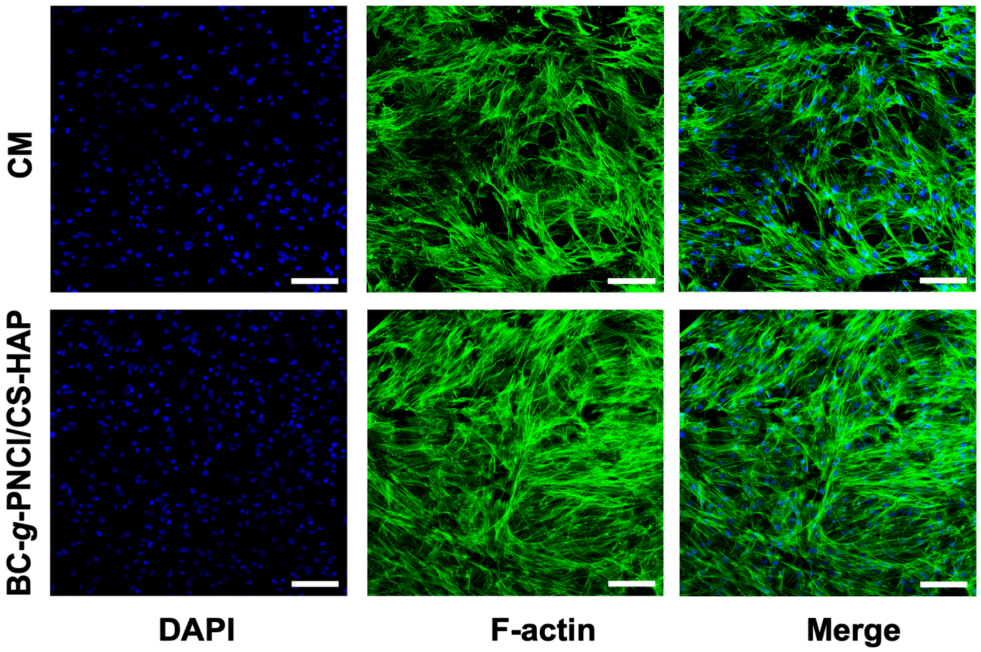

Supplementary Fig. 6 Morphology, extension, and viability of BMSCs. Morphology of BMSCs cultured on loose layer of CM and CS-HAP layer of BC-*g*-PNCl/CS-HAP at the 7th day (green for F-actin, blue for cell nucleus, scale bars =100 μm). Fluorescent observation was repeated three times independently, yielding similar results.

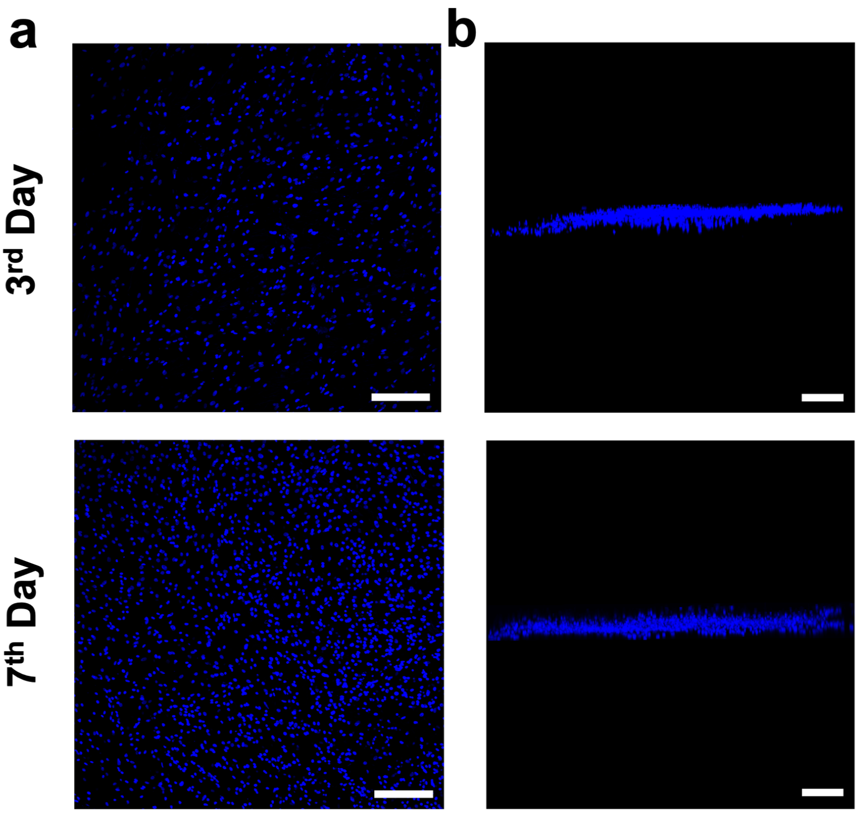

Supplementary Fig. 7 Proliferation and distribution of BMSCs. a, b Top-view **(a)** and cross-section **(b)** fluorescent images exhibiting the proliferation area and depth of BMSCs cultured on CS-HAP layer of BC-*g*-PNCl/CS-HAP at the 3rd and 7th days, respectively (blue for cell nucleus, scale bars = 200 μm). Fluorescent observation was repeated three times independently, yielding similar results.

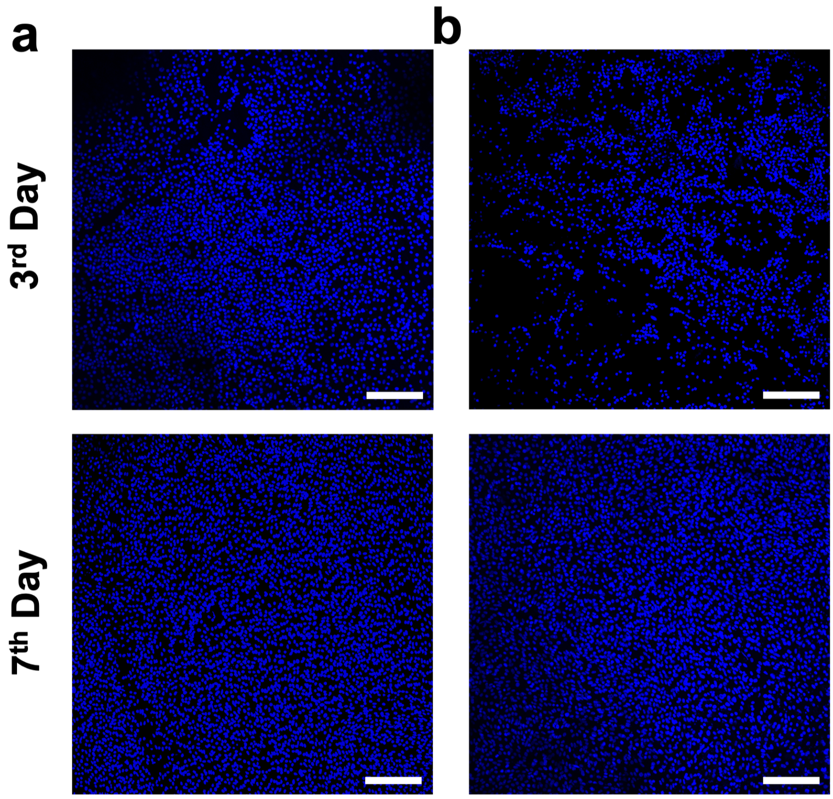

Supplementary Fig. 8 Proliferation and distribution of L929s. Top-view fluorescent images exhibiting the proliferation area of L929s cultured on dense layer of CM **(a)** and BC-*g*-PNCl layer of BC-*g*-PNCl/CS-HAP **(b)** at the 3rd and 7th days, respectively (blue for cell nucleus, scale bars = 200 μm). Fluorescent observation was repeated three times independently, yielding similar results.

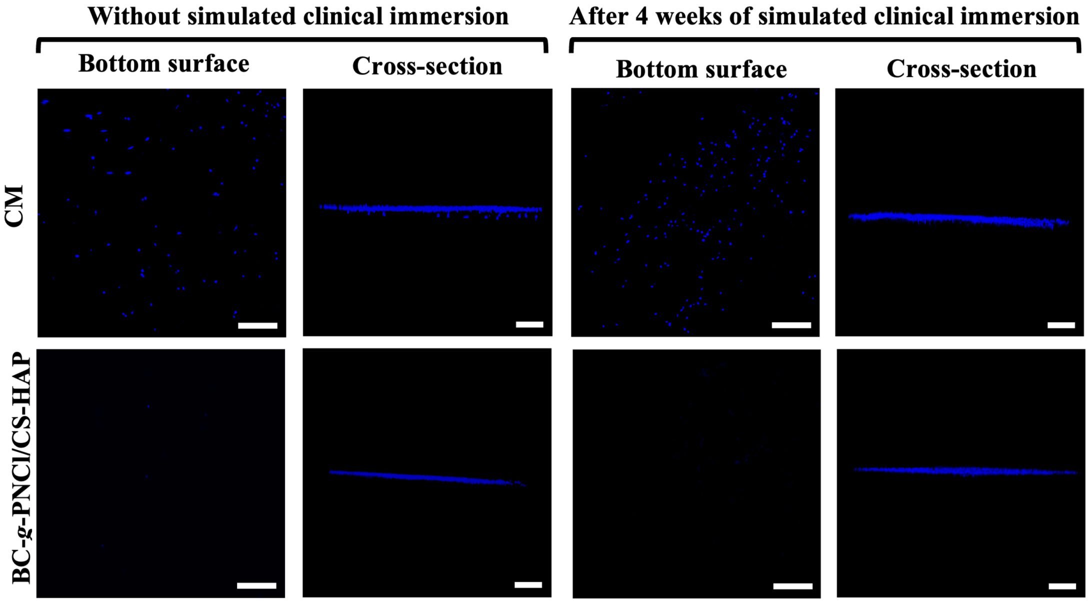

Supplementary Fig. 9 Barrier function against L929s. Fluorescent images of penetrated cells on the bottom surfaces and penetration depths after 3 days for CM and BC-*g*-PNCl/CS-HAP, exhibiting their barrier functions against L929s before and after simulated clinical immersion for 4 weeks (blue for cellular nucleus, scale bars = 200 μm). Fluorescent observation was repeated three times independently, yielding similar results.

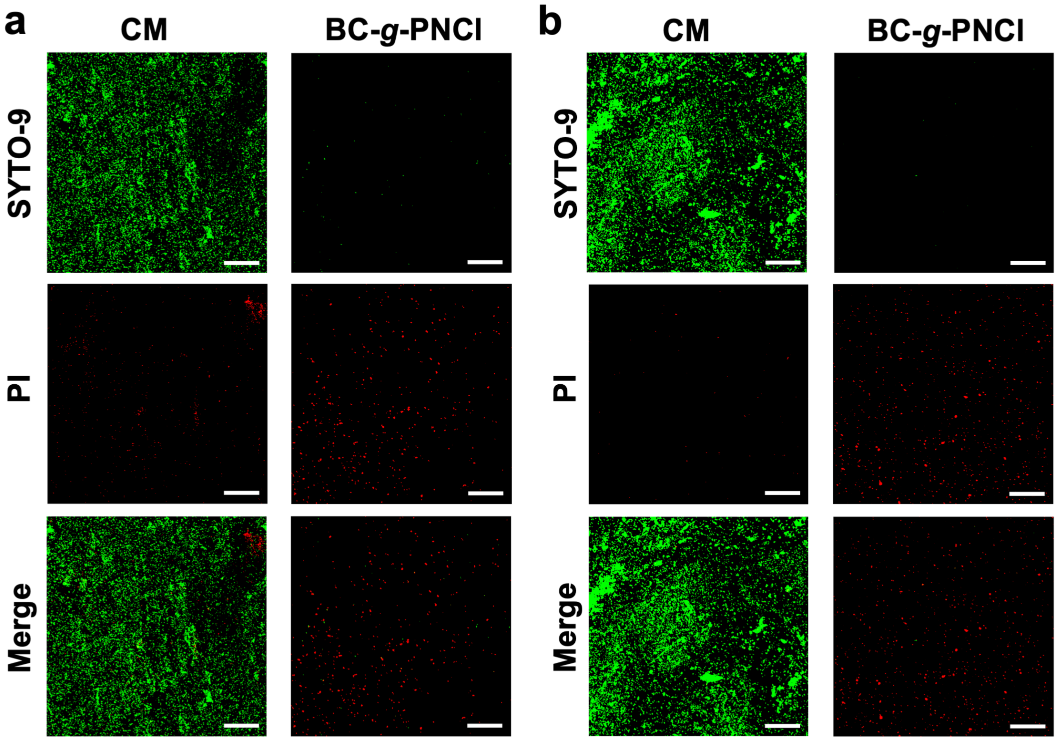

Supplementary Fig. 10 Anti-adhesion and anti-bacterial performances. **a, b** Fluorescent images exhibiting the live/dead bacterial distributions of *S. aureus* **(a)** and *P. gingivalis* **(b)** on the surfaces of CM and BC-*g*-PNCl. Only sporadic dead bacteria exist on the surface of BC-*g*-PNCl, while plenty of live bacteria are bred on the surface of CM (green for live bacteria, red for dead bacteria, scale bars = 50 μm). Fluorescent observation was repeated three times independently, yielding similar results.

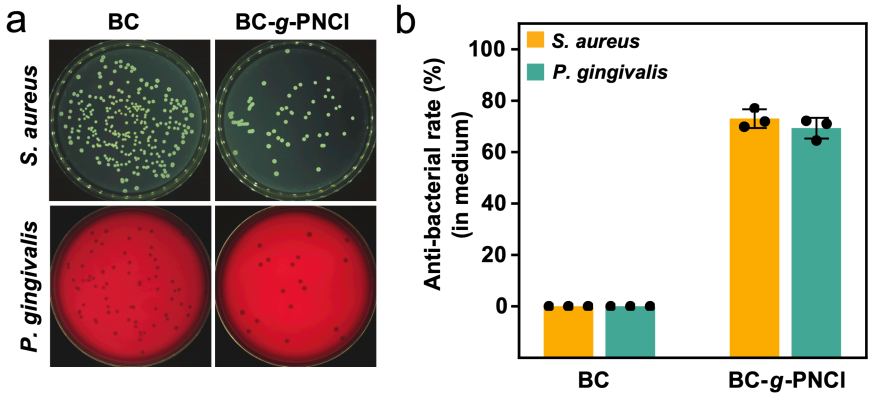

Supplementary Fig. 11 Release-killing ability. **a** Images of the bacterial colonies formed by *S. aureus* and *P.gingivalis* in the medium treated with BC and BC-*g*-PNCl. **b** Release-killing rates of BC and BC-*g*-PNCl against *S. aureus* and *P. gingivalis* according to **a** (n = 3 independent samples; error bars = SD; data are presented as mean values ± SD).

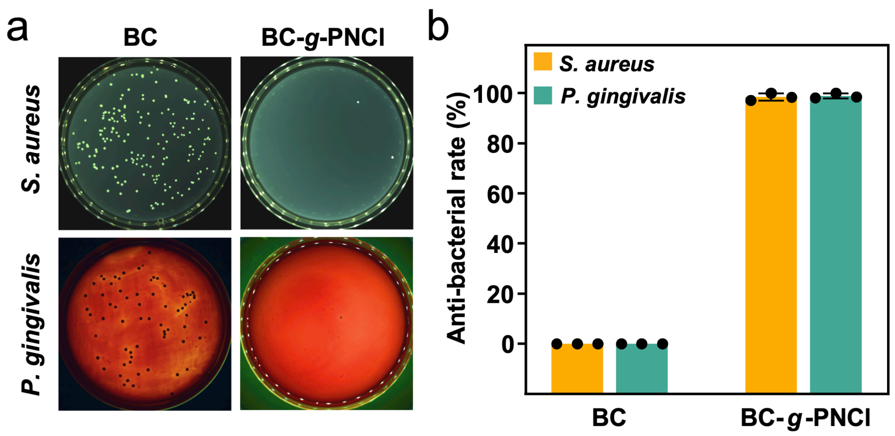

Supplementary Fig. 12 Contact-killing ability. **a** Images of the bacterial colonies formed by *S. aureus* and *P. gingivalis* contacted with BC and BC-*g*-PNCl. **b** Contact-killing rates against *S. aureus* and *P. gingivalis* contacted with BC and BC-*g*-PNCl according to **a** (n = 3 independent samples; error bars = SD; data are presented as mean values ± SD).

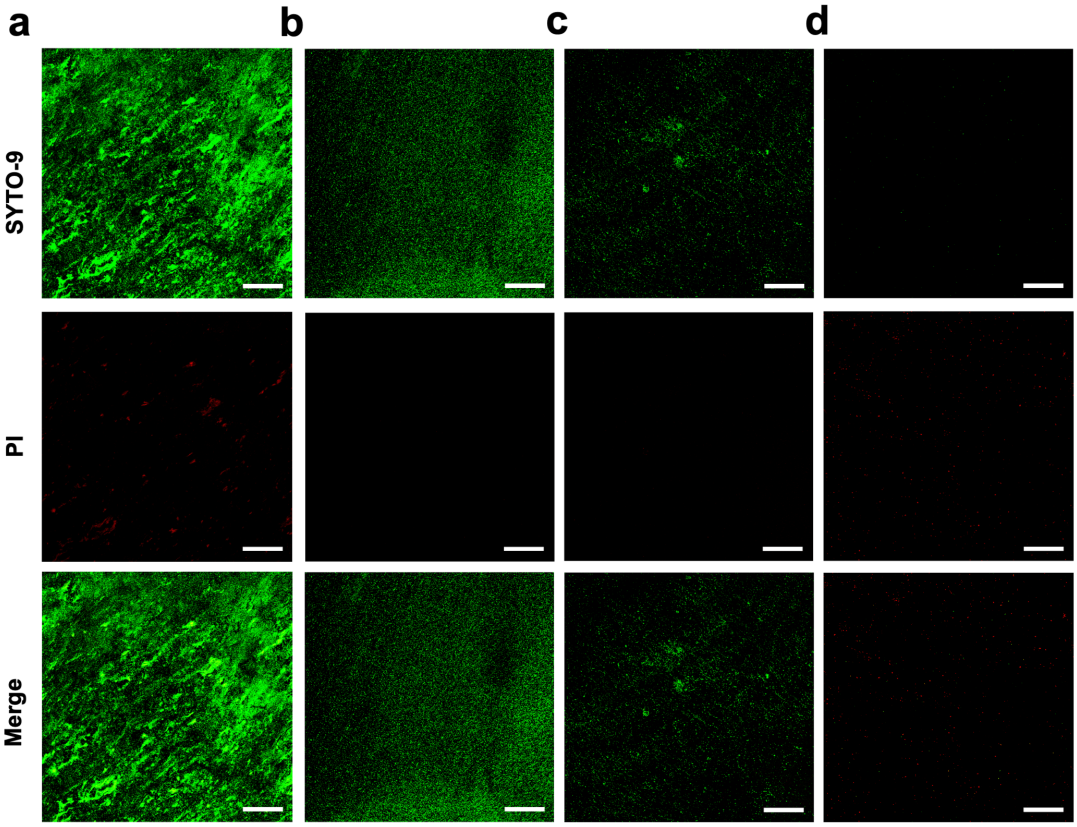

Supplementary Fig. 13 Anti-adhesion ability. Fluorescent images exhibiting bacterial adhesion of *S. aureus* on the surfaces of CM dense layer (**a**), BC (**b**), BC-*g*-PAM (**c**), and BC-*g*-PNCl (**d**) (green for live bacteria , red for dead bacteria , scale bars = 200 μm). Fluorescent observation was repeated three times independently, yielding similar results.

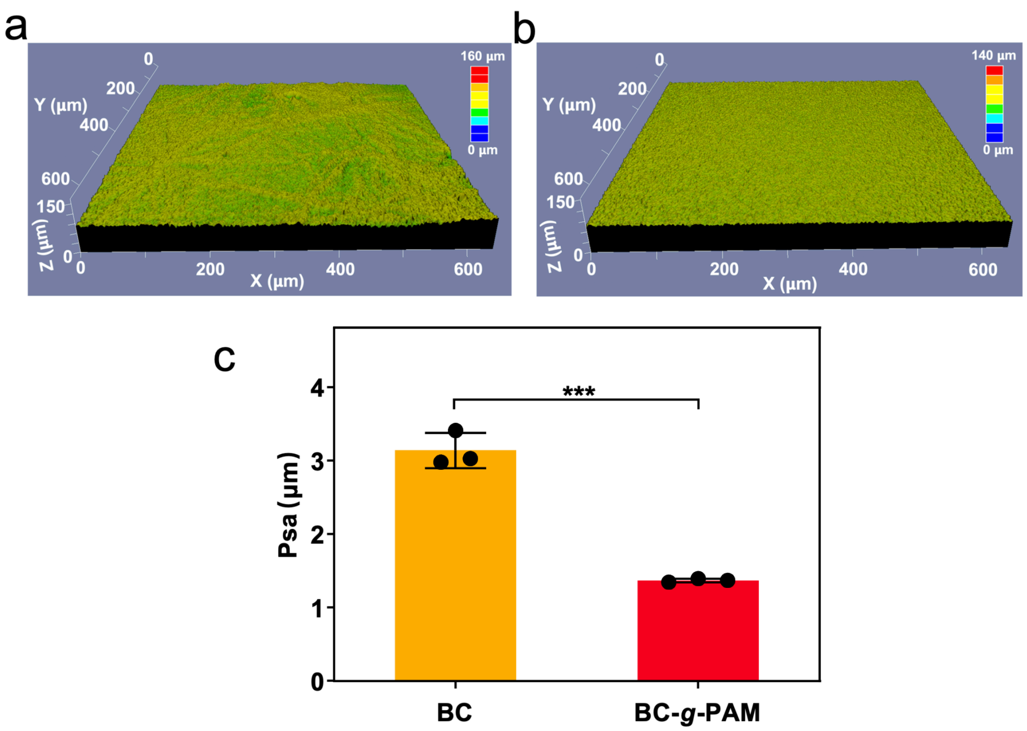

Supplementary Fig. 14 Surface roughness of BC and BC-*g*-PAM. a, b Roughness of BC (**a**) and BC-*g*-PAM (**b**). **c** Roughness analysis between BC and BC-*g*-PAM (n = 3 independent samples; Student's t test; *** two-tailed $p = 0.0002$; error bars = SD; data are presented as mean values ± SD).

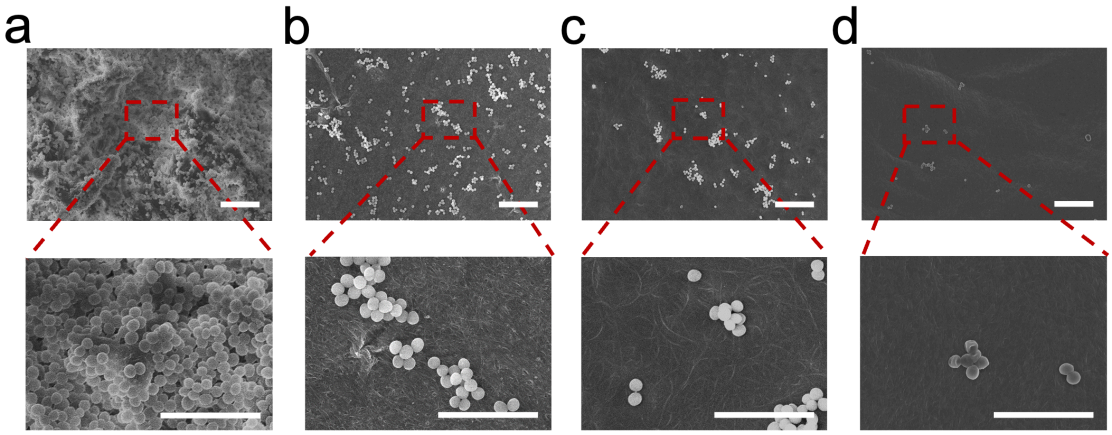

Supplementary Fig. 15 Anti-adhesion and anti-biofilm capacities. a-d SEM images exhibiting the bacterial adhesion situation of *S. aureus* on the surfaces of CM **(a)**, BC **(b)**, BC-*g*-PAM **(c)**, and BC-*g*-PNCl **(d)**. The number of bacteria on the surfaces of the CM dense layer, BC, BC-*g*-PAM, and BC-*g*-PNCl is gradually reduced (upper scale bars = 10 μm, lower scale bars = 5 μm) . SEM observation was repeated three times independently, yielding similar results.

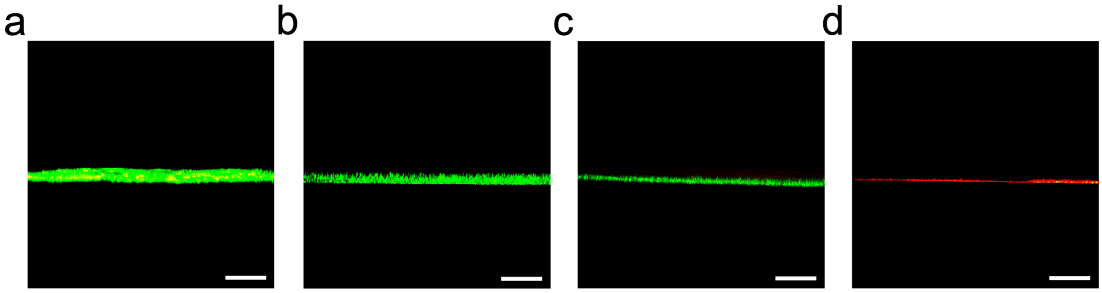

Supplementary Fig. 16 Anti-biofilm capacities. Three-dimensional fluorescent images exhibiting the bacterial adhesion situation of *S. aureus* on the surfaces of CM **(a)**, BC **(b)**, BC-*g*-PAM **(c)**, and BC-*g*-PNCl **(d)**. The depth of biofilm on the surfaces of the CM dense layer, BC, BC-*g*-PAM, and BC-*g*-PNCl is gradually reduced (green for live bacteria, red for dead bacteria, scale bars = 200 μm). Fluorescent observation was repeated three times independently, yielding similar results.

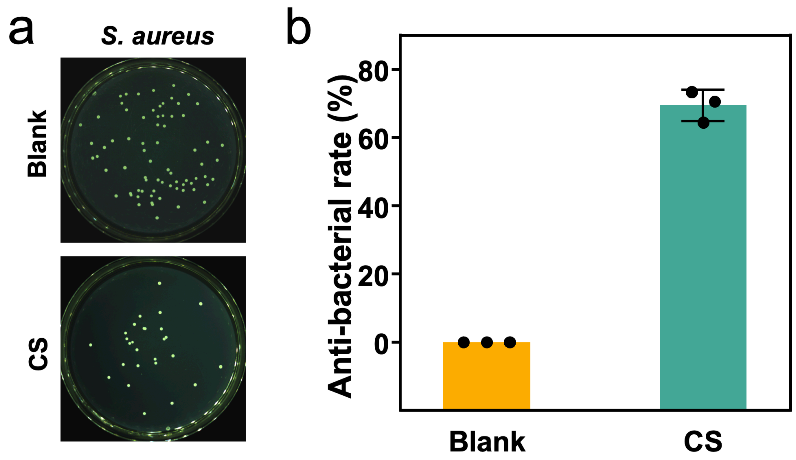

Supplementary Fig. 17 Anti-bacterial ability of CS. **a** Images of the bacterial colonies formed by *S. aureus* in the medium in the blank group and CS group. **b** Anti-bacterial rate of CS against *S. aureus* according to **a** (n = 3 independent samples; error bars = SD; data are presented as mean values ± SD).

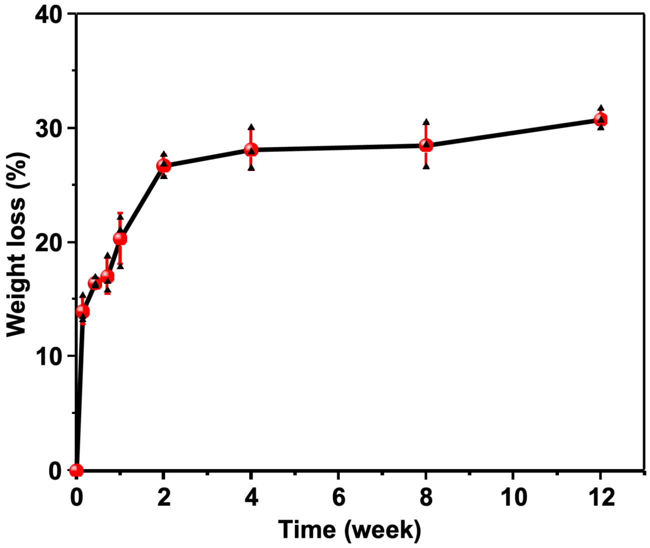

Supplementary Fig. 18 Degradation of BC-*g*-PNCl/CS-HAP after incubation in PBS for 12 weeks (n = 3 independent samples; error bars = SD; data are presented as mean values ± SD).

Supplementary Table 1 Primer sequences used for RT-qPCR

Genes	Primers
ALP	Forward: 5' - GCACAACATCAAGGACATCG - 3'<br>Reversed: 5' - TCAGTTCTGTTCTTGGGGTACAT - 3'
Col1	Forward: 5' - GGTTTGGAGAGAGCATGACC - 3'<br>Reversed: 5' - TTTGGGGAAATTGAGTTTGG - 3'
RUNX2	Forward: 5' - GCGGTGCAAACTTTCTCCAG - 3'<br>Reversed: 5' - TGCAGCCTTAAATGACTCGG - 3'
OCN	Forward: 5' - CCCAATTGTGACGAGCTAGC - 3'<br>Reversed: 5' - CTGTGCCGTCCATACTTTCG - 3'
GAPDH	Forward: 5' - TCTCTGCTCCTCCCTGTTCT - 3'<br>Reversed: 5' - CCGATACGGCCAAATCCGTT - 3'
