# Supplementary material for: All-in-one porous membrane enables full protection in guided bone regeneration
Source: Nat Commun. 2024 Jan 2;15:119. doi: 10.1038/s41467-023-43476-9 (PMC10762214; doi:10.1038/s41467-023-43476-9)
Supplement: Supplementary file 3 — Description of Additional Supplementary Files [file 41467_2023_43476_MOESM3_ESM.pdf]

Title: Supplementary Movie 1:
Description: BC-g-PNCl/CS-HAP can be maintained under high-speed vibration, indicating its tightly bound bilayer structure

Title: Supplementary Movie 2:
Description: The wet BC-g-PNCl/CSHAP only slightly deforms when the loading (1 g) is applied and quickly returns to its original form after removing the loading.

Title: Supplementary Movie 3:
Description: The wet CM collapses completely when the loading (1 g) is applied and remains irreversible after removing the loading.
